# Prevalence of Body Dysmorphic Disorder in the Spanish Population: Cross-Sectional Web-Based Questionnaire Study

**DOI:** 10.2196/46515

**Published:** 2024-01-04

**Authors:** Álvaro Loewen, Hilario Blasco-Fontecilla, Chao Li, Marcos Bella-Fernández, Belén Ruiz-Antorán

**Affiliations:** 1 Facultad de Medicina Universidad Autónoma de Madrid Madrid Spain; 2 Servicio de Psiquiatría Infanto-juvenil Hospital Universitario Puerta de Hierro Majadahonda Majadahonda Spain; 3 Centro de Investigación Biomédica en Red - Salud Mental Madrid Spain; 4 Facultad de Ciencias de la Salud y Centro Médico Universidad Internacional de La Rioja Madrid Spain; 5 Facultad de Psicología Universidad Autónoma de Madrid Madrid Spain; 6 Departamento de Psicología Universidad Pontificia de Comillas Madrid Spain; 7 Servicio de Farmacología Clínica Hospital Universitario Puerta de Hierro Majadahonda Majadahonda Spain

**Keywords:** body dysmorphic disorder, prevalence, adults, Spain, comorbidities, mental health, depression, anxiety, OCD, obsessive-compulsive disorder

## Abstract

**Background:**

Body dysmorphic disorder (BDD) is defined as excessive concern with mild or nonexistent defects in personal physical appearance, which are not perceived by others. The worldwide prevalence of BDD ranges between 0.5% and 3.2%, with no differences across genders. The mean age of onset of BDD is 16.9 years. BDD is typically associated with young age, psychiatric disorders, and dermatological procedures. Patients with BDD typically display poorer mental health status than patients diagnosed with other mental disorders.

**Objective:**

The aim of this study was to estimate the prevalence of BDD in Spain and to identify the variables associated with BDD.

**Methods:**

We performed a cross-sectional descriptive study by collecting data through an anonymous web-based survey targeting the Spanish population aged 18 years or older. The measures in this study were (1) sociodemographic variables, (2) variables associated with dermatological and psychiatric disorders and cosmetic procedures, (3) scales measuring quality of life (12-item Short Form health survey, version 2) and (4) BDD (BDD Questionnaire). Statistical analysis was performed with SPSS software version 21. *P* values less than .05 were considered significant.

**Results:**

Of the 2091 participants who took the survey, 322 (15.2%) met the criteria of having BDD. The mean age of the participants with BDD was 23.5 (SD 9.6) years. In terms of BDD prevalence, women accounted for 19.9% (284/1421), men accounted for 5.2% (34/653), and students accounted for 25.2% (263/1043). Approximately 46.6% (150/322) of the participants with BDD reported a history of psychiatric comorbidities, including anxiety disorders, depressive disorders, and eating disorders. BDD was significantly associated with female gender, younger age (18-24 years), students, monthly income of less than €500 (€1=US $1.11), and the presence of dermatological and some psychiatric disorders such as depression, anxiety, and eating disorders (*P*<.05). The number of body parts of concern in participants with BDD was significantly higher than that in those without BDD (4.6 vs 2.2, respectively; *P*<.001). Regarding the body parts of concern, body fat was the most common concern for both groups with BDD and without BDD, followed by thighs, face, hips, and skin in the BDD group and thighs, teeth, and hair in the non-BDD group. Participants with BDD showed a significantly poorer self-perception of their mental health, irrespective of the presence of any mental disorder (*P*<.001).

**Conclusions:**

Our findings showed that the prevalence of BDD in Spain was higher than expected. Further, BDD is frequently associated with other psychiatric disorders, particularly depressive disorder, anxiety disorder, and eating disorder. Participants with BDD had a poorer perception of quality of life associated with mental but not physical health problems. Finally, the perception of quality of mental health life in participants with BDD was independent of diagnosis of any mental disorder.

## Introduction

Body dysmorphic disorder (BDD) is a common psychiatric disorder affecting 0.5%-3.2% of the general population worldwide [1]. A multicentric study in Spain showed that the prevalence of BDD was higher among patients with acne (10.6%) [2]. The prevalence of BDD across genders is debatable. One study showed similar prevalence of BDD across both genders (49% females and 51% males) [3]. Another study reported higher prevalence among females (68.5%) [4]. The average age of onset of BDD is 16.9 years [5]. One study reported an inverse relationship between the prevalence of BDD and age: 78.6% of participants with BDD were aged 18-27 years, 14.3% were aged 28-37 years, and 7.1% were aged 38 years or older [6]. Furthermore, BDD is closely associated with other mental disorders. A recent systematic review [1] showed that the highest prevalence of BDD was among psychiatric inpatients (5.8%-37.78%). The psychiatric disorders most frequently associated with BDD are depressive disorder (47%-56.3%), borderline personality disorder (54.3%), and eating disorders (12%-45%), whereas obsessive-compulsive disorder (3%-15.3%) and schizophrenia are less closely associated with BDD. Participants who had cosmetic procedures (2.9%-57%) slightly overlay with BDD prevalence in the general population. The prevalence of BDD in both psychiatric outpatients (0.3%-2%) and students (1.3%-5.8%) partially overlaps with that observed in the general population. Among dermatologic patients, the prevalence of BDD was reported to be 2.1%-36% [1].

The etiology of BDD is multifactorial, encompassing biological, psychological, and sociocultural factors. BDD has been associated with parental rejection, as well as physical, emotional, or sexual abuse during adolescence [7]. Studies have shown a possible genetic association in first-degree relatives, with affected patients being up to 3-8 times more likely to develop BDD than the general population [7]. Shy, anxious, and perfectionistic individuals may also have a greater predisposition to develop this disorder [8].

The most important symptom of BDD is distortion of body perception, which leads to low self-esteem, anxiety, depression, social isolation, and obsessive-compulsive behaviors [9]. The clinical profile of BDD is characterized by repetitive actions such as constant checking in mirrors, applying excessive makeup to cover defects, dermatillomania, comparing one's appearance with that of others, and excessive exercise, taking up an average of 3-8 hours daily [10]. Patients with BDD are usually preoccupied with 5-7 different parts of their body [11], the most common being skin (53.8%), nose (38.5%), and hair (34.6%). The other body parts that are of frequent concern are weight and muscle mass (30.8%), face (30.8%), chest and trunk (19.2%), and teeth (19.2%) [1]. The mental and physical health status perceived by patients with BDD are lower than that perceived by the general population [12].

Given that the primary concern of patients with BDD revolves around their external appearance, it is common for people with BDD to predominantly seek dermatological and cosmetic procedures over seeking professional help for the treatment of their underlying psychiatric pathology [13]. Moreover, patients diagnosed with BDD often do not seek help for various reasons: they feel ashamed or lack insight [14]. Furthermore, BDD is likely to be underdiagnosed, given the large number of barriers to diagnosis such as the absence of appropriate tools, lack of information and awareness among health care professionals, and professionals' refusal to lose a patient or inability to diagnose it properly [15]. In addition, not all health care professionals are familiar with this disorder [9]. This leads to poor identification in psychiatric and cosmetic settings where BDD cases are notoriously prevalent [14]. If professionals do not perform a detailed anamnesis, it is difficult for patients to disclose their concerns, given the shyness underlying this disorder [16]. To overcome this shortcoming, clinicians may use the BDD Questionnaire (BDDQ), a validated diagnostic tool for BDD, with sensitivity of 100% [17-19], specificity of 89%-93% for psychiatric inpatients [18], and 93% for dermatologic patients [19]. The BDDQ is a brief questionnaire that assesses different items of the patient’s body perception [17]. Finally, treatment is based on pharmacological and nonpharmacological measures. The former includes the use of fluoxetine, a serotonin reuptake inhibitor antidepressant. The latter is based on psychotherapy—most notably cognitive behavioral therapy [20]. Randomized clinical trials have shown a rate of 50%-80% improvement in patients as well as a lower relapse rate following pharmacological treatment [21].

In summary, BDD is a poorly studied and underdiagnosed psychiatric disorder. This may be because BDD is not perceived as a disorder by aesthetic practitioners, who may think that they are merely offering a “service” [22-24]. The main objective of our study was to estimate the prevalence of BDD in Spain. Additionally, we explored the association of BDD with sociodemographic variables, presence of dermatological or psychiatric disorders and cosmetic procedures, and quality of life.

## Methods

### Design and Scope of This Study

A cross-sectional descriptive study was conducted. The general population aged 18 years and older in Spain was invited to access the study protocol via a link to Google Forms. The security and lawful use of personal data collected on the website is guaranteed by accepting the data privacy policy included in the survey. The measures in the protocol consisted of (1) sociodemographic variables, (2) variables associated with dermatological and psychiatric disorders and cosmetic procedures, and (3) perception of health and quality of life, measured using the 12-item Short Form version 2 (SF-12v2) health survey, and (4) a validated scale for the diagnosis of BDD, using the BDD questionnaire screening test [17].

### Study Sample Population

Participants 18 years and older from the general population residing and registered in Spain at the time the survey was performed and who voluntarily completed the study's web-based questionnaire were included. The sample size was calculated using Epidat 4.0 (Dirección Xeral de Saúde Pública da Consellería de Sanidade da Xunta de Galicia) based on the following estimate: population size, 40,000,000; expected proportion, 5%; accuracy, 1%; confidence level, 95%; and design effect, 1.0. The minimum total number of responses required for 1% precision with 95% confidence level was 1825 participants.

### Variables

The following variables were collected: sociodemographic variables such as gender (female, male, or other, ie, participants who do not identify themselves as male or female), year of birth, region of residence, race, educational level, employment status, and range of monthly income. Variables related to other comorbidities associated with BDD were comorbidity with dermatologic and psychiatric diseases and cosmetic procedures. Regarding the year of birth, the participants were classified into 4 age groups: 18-24 years, 25-44 years, 45-64 years, and 65 years or older. This classification was based on previous studies related to mental health and the use of these services according to age group [25].

### Quality of Life Assessment

Data on quality of life and perception of their state of health were collected through the SF-12v2 health survey, which is validated as a psychometric instrument for numerous diseases and conditions, including mental illness. It assesses the participant's physical and mental state through 8 health domains: 4 related to physical health, that is, general health, physical function, role-physical, and bodily pain; and 4 related to mental health, that is, vitality, social function, role-emotional, and mental health [26].

### BDD Assessment

BDD assessment was performed using the BDDQ [17]. A version adapted and validated in Spanish was used [27]. The BDDQ is a brief (7-item) self-report measure derived from the Diagnostic and Statistical Manual of Mental Disorders, fourth edition diagnostic criteria. The first 6 items require a dichotomous answer (yes/no) and the last one is multiple choice. The test will be positive if the patient answers “yes” to questions 1 and 2, “yes” to question 3, 4, 5, or 6, and if in question 7, the time indicated is more than 1 hour per day [17].

### Data Handling

The Google Forms platform was used for the web-based survey. The questionnaire was distributed telematically, both through a link via mobile phone and a printed QR code. The data obtained were extracted and sorted in Microsoft Excel. Subsequently, we used SPSS to create the database and perform the corresponding analyses. A license was obtained for the SF-12v2 health survey, which together with the use of the QualityMetric program provided, allowed for its correct interpretation. The database was generated in an anonymized form guaranteeing exclusive access by the principal investigator, thereby allowing respect, privacy, and confidentiality of the data.

### Statistical Analysis

Statistical analysis was performed with SPSS software (version 21; IBM Corp). All statistical analyses were performed at .05 level of significance. A descriptive study was conducted for all the variables included in this study. Quantitative variables were expressed as mean and standard deviation (SD). Qualitative variables were expressed as absolute value (n) and percentage with an estimated 95% CI. Comparison of means was performed using 2-sided Student *t* test or Mann-Whitney *U* test as appropriate after checking normality with the Kolmogorov-Smirnov test. The association of the qualitative variables was estimated by means of the chi-square statistic. Multiple logistic regression models were used to determine the association of different variables with each other. A univariate analysis was performed where a significantly higher risk ratio for BDD diagnosis was obtained for some of the variables studied. The significant variables obtained in the univariate model were subsequently included in the multivariate analysis. Thereafter, given the heterogeneous conditions of the population, a subgroup analysis was performed with a multivariate model considering the gender and age variables.

### Ethics Approval

This study was conducted in accordance with the requirements expressed in the Declaration of Helsinki 2013. Participants were invited to participate online by clicking on the link to the survey. Information about the purpose of the survey and its anonymous and voluntary nature was included in the survey header. Participants were identified by a numerical code in order to respect the confidentiality of the participants' personal data. The automatic code is assigned directly by Google Forms at the time of download through a time stamp. This project was approved by the ethics committee of the Hospital Universitario Puerta de Hierro Majadahonda in Madrid (promoter protocol code PI 206/21, December 20, 2021).

## Results

A total of 2091 participants were included in this study. The sociodemographic and clinical characteristics of the participants are described in Table 1.

**Figure 1:**
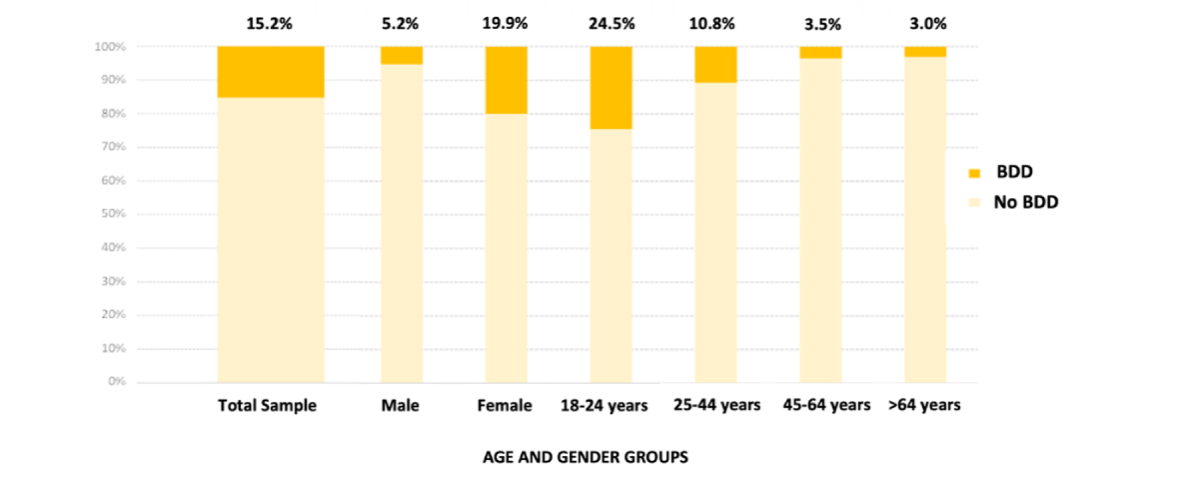
Prevalence of body dysmorphic disorder among subsamples. BDD: body dysmorphic disorder.

**Figure 2:**
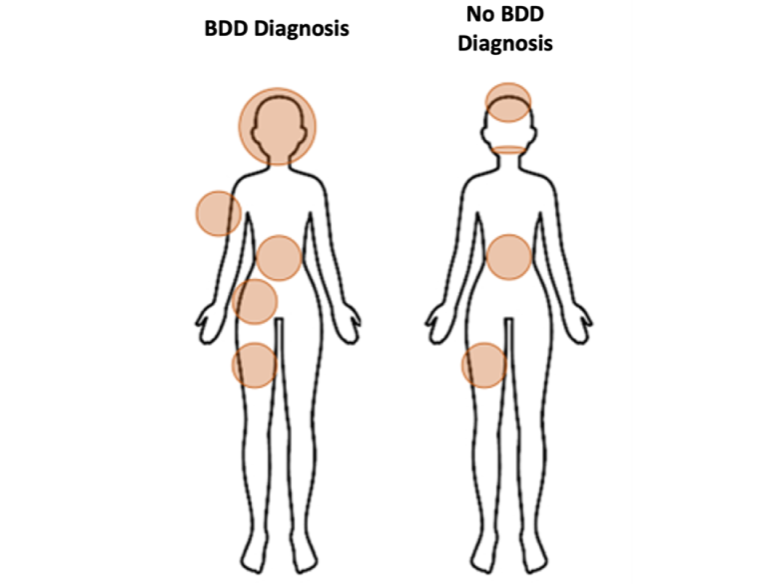
Body parts of concern in our study population.

The prevalence of BDD in the population assessed in this study was 15.2% (284/1421, 19.9% in females vs 34/653, 5.2% in males). Regarding age, the prevalence of BDD was higher in the youngest age group (18-24 years; 267/1091, 24.5%), followed by the 25-44 years age group (30/279, 10.8%) (Figure 1). The number of body parts of concern in participants with BDD was significantly higher than that in participants without BDD (4.6 vs 2.2, respectively; *P*<.001). Regarding the body parts of concern, body fat was the most common concern in both groups, followed by thighs, face, hips, and skin in the BDD group and thighs, teeth, and hair in the non-BDD group (Figure 2). Regarding the sociodemographic characteristics of the BDD population (n=322), the majority were females (284/322, 88.2%), with a mean age of 23.5 (SD 9.6) years, and Caucasians (243/722, 75.5%). Approximately 81.7% (1533/2091) were students, and 76.6% (247/322) of them had a university education level. Among the participants diagnosed with BDD, 63.4% (204/322) had a history of dermatologic disease, the most frequent being acne (115/204, 56.5%) and dermatitis (99/204, 48.5%). Approximately 46.6% (150/322) of the participants with BDD reported a history of psychiatric comorbidities, and the most frequent were anxiety disorders (108/150, 72%), depressive disorders (84/150, 56%), eating disorders (72/150, 48%), and attention-deficit/hyperactivity disorder (18/150, 12%). Approximately 17.7% (57/322) of the population with BDD had previously undergone a cosmetic procedure, the most frequent being laser treatment for acne, blemishes, and other skin lesions (17/57, 29.8%); mesotherapy (9/57, 15.8%); and rhinoplasty (7/57, 12.3%). The factors related to BDD are reported in univariate and multivariate analyses in Table 2. BDD diagnosis was significantly associated with female gender, other genders, age 18-24 years, students, monthly income level of less than €500 (€1=US $1.11), and participants with dermatologic and psychiatric comorbidities (*P*<.001). All these variables were included in the multivariate model, with gender (female and other), age, student occupation, depressive disorder, eating disorders, and anxiety disorders remaining at <.05 significance as diagnostic predictors of BDD.

**Table 1:** Sociodemographic and clinical characteristics of the participants.

		All (N=2091)	With BDD^a^ diagnosis (n=322)	Without BDD diagnosis (n=1769)
Gender (female), n (%)		1421 (68)	284 (88.2)	1137 (64.3)
Age (years), mean (SD)		37.7 (16.6)	23.5 (9.6)	35.5 (16.9)
**Age group (years), n (%)**				
	18-24	1091 (52.2)	267 (82.9)	824 (46.6)
	25-44	279 (13.3)	30 (9.3)	249 (14.1)
	45-64	654 (31.3)	23 (7.1)	631 (35.7)
	>64 years	66 (3.2)	2 (0.6)	64 (3.6)
**Ethnicity, n (%)**				
	African	11 (0.5)	3 (0.9)	8 (0.5)
	American	6 (0.3)	0 (0)	6 (0.3)
	Asian	26 (1.2)	7 (2.2)	19 (1.1)
	Caucasian	1533 (73.3)	243 (75.5)	1290 (72.9)
	Latin	306 (14.6)	23 (7.1)	283 (16)
	Other	209 (10)	46 (14.3)	163 (9.2)
**Educational level, n (%)**				
	Elementary school	11 (0.5)	1 (0.3)	10 (0.6)
	Middle school	8 (0.4)	0 (0)	8 (0.5)
	Professional education	100 (4.8)	15 (4.7)	85 (4.8)
	High school	265 (12.7)	59 (18.3)	206 (11.6)
	University	1707 (81.6)	247 (76.7)	1460 (82.5)
**Occupation, n (%)**				
	Student	1043 (49.9)	263 (81.7)	780 (44.1)
	Worker	865 (41.4)	50 (15.5)	815 (46.1)
	Other	183 (8.8)	9 (2.8)	174 (9.8)
**Monthly income (€)^b^, n (%)**				
	<500	861 (41.2)	205 (63.7)	656 (37.1)
	500-999	138 (6.6)	34 (10.6)	104 (5.9)
	1000-1999	343 (16.4)	39 (12.1)	304 (17.2)
	2000-2999	291 (13.9)	9 (2.8)	282 (15.9)
	>3000	358 (17.1)	11 (3.4)	347 (19.6)
	Not known	100 (4.8)	24 (7.5)	76 (4.3)
**Dermatologic disease, n (%)**		1072 (51.3)	204 (63.4)	868 (49.1)
	Acne	532 (49.6)	115 (56.5)	417 (48)
	Atopic dermatitis	434 (40.5)	99 (48.5)	335 (38.6)
	Other dermatitis	35 (3.3)	10 (4.9)	25 (2.9)
	Psoriasis	50 (4.7)	2 (1)	48 (5.5)
	Rosacea	64 (6)	5 (2.5)	59 (6.8)
	Urticaria	15 (1.4)	1 (0.5)	14 (1.6)
	Pityriasis	10 (0.9)	1 (0.5)	9 (1)
	Eczema	37 (3.5)	9 (4.4)	28 (3.2)
	Skin infections	32 (3)	6 (2.9)	26 (3)
	Neoplasms	11 (1)	0 (0)	11 (1.3)
	Other	65 (6.1)	7 (3.4)	58 (6.7)
**Psychiatric disorder, n (%)**		493 (23.6)	150 (46.6)	343 (19.4)
	Depressive Disorder	244 (49.5)	84 (56)	160 (46.6)
	Borderline personality disorder	9 (1.8)	3 (2)	6 (1.7)
	Eating disorder	136 (27.6)	72 (48)	64 (18.7)
	Obsessive-compulsive disorder	33 (6.7)	12 (8)	21 (6.1)
	Attention-deficit/hyperactivity disorder	59 (12)	18 (12)	41 (12)
	Bipolar disorder	7 (1.4)	1 (0.7)	6 (1.7)
	Anxiety disorder	292 (59.2)	108 (72)	184 (53.6)
	Schizophrenia/psychosis	0 (0)	0 (0)	0 (0)
	Substance abuse	12 (2.4)	3 (2)	9 (2.6)
	Other	18 (3.7)	3 (2)	15 (4.4)
History of plastic surgery procedures, n (%)		355 (17)	57 (17.7)	298 (16.8)

^a^BDD: body dysmorphic disorder.

^b^€1=US $1.11.

**Table 2:** Factors related to body dysmorphic disorder.

Factor				Univariate risk ratio (95% CI)		*P* value		Multivariate risk ratio (95% CI)		*P* value
**Gender**										
	Female	284/1421 (20)	4.55 (3.14-6.58)		.001		3.02 (2.01-4.53)		.001	
	Male	34/653 (5.2)	Ref^a^		—^b^		—		—	
	Other	4/17 (23.5)	5.60 (1.73-18.09)		.004		4.57 (1.28-16.38)		.02	
Mean age				0.93 (0.92-0.95)		.001		0.97 (0.95-0.99)		.02
**Age categories (years)**										
	18-24	267/1091 (24.5)	2.69 (1.79-4.02)		.001		—^b^		—	
	25-44	30/279 (10.8)	Ref		—		—		—	
	45-64	23/654 (3.5)	0.30 (0.17-0.53)		.001		—		—	
	>64	2/66 (3)	0.26 (0.060-1.11)		.07		—		—	
**Occupation**										
	Student	263/1043 (25.2)	Ref		—		—		—	
	Worker	50/865 (5.8)	0.18 (0.13-0.25)		.001		0.51 (0.28-0.94)		.03	
	Other	9/183 (4.9)	0.15 (0.07-0.30)		.001		0.35 (0.14-0.88)		.03	
**Dermatologic disease**										
	Yes	204/1072 (19)	1.79 (1.40-2.29)		.001		—		—	
	No	118/1019 (11.6)	—		—		—		—	
**Acne**										
	Yes	115/532 (21.6)	1.80 (1.40-2.32)		.001		—		—	
	No	207/1559 (13.3)	—		—		—		—	
**Atopic dermatitis**										
	Yes	99/434 (22.8)	1.90 (1.46-2.48)		.001		—		—	
	No	223/1657 (13.5)	—		—		—		—	
**Other dermatitis**										
	Yes	10/35 (28.6)	2.23 (1.06-4.70)		.03		—		—	
	No	312/2056 (15.2)	—		—		—		—	
**Psoriasis**										
	Yes	2/50 (4)	0.22 (0.05-0.92)		.02		—		—	
	No	320/1041 (15.7)	—		—		—		—	
**Psychiatric disorder**										
	Yes	150/493 (30.4)	3.63 (2.83-4.65)		.001		—		—	
	No	172/1598 (10.8)	—		—		—		—	
**Depressive Disorder**										
	Yes	84/244 (34.4)	3.55 (2.64-4.78)		.001		1.68 (1.06-2.69)		.03	
	No	238/1847 (12.9)	—		—		—		—	
**Eating behavior disorder**										
	Yes	72/136 (52.9)	7.67 (5.34-11.02)		.001		3.77 (2.26-6.30)		.001	
	No	250/1955 (12.8)	—		—		—		—	
**Obsessive-compulsive disorder**										
	Yes	12/33 (36.4)	3.22 (1.57-6.62)		.001		—		—	
	No	310/2058 (15.1)	—		—		—		—	
**Attention-deficit/hyperactivity disorder**										
	Yes	18/59 (30.5)	2.49 (1.41-4.40)		.003		—		—	
	No	304/2032 (15)	—		—		—		—	
**Anxiety disorder**										
	Yes	108/292 (37)	4.35 (3.30-5.74)		.001		2.30 (1.36-3.88)		.002	
	No	214/1799 (11.9)	—		—		—		—	
**Plastic surgery**										
	Yes	57/355 (16.1)	1.06 (0.78-1.45)		.71		—		—	
	No	298/355 (83.9)	—		—		—		—	

^a^Ref: reference value.

^b^Not applicable.

Given the heterogeneity of the population, most of whom were women aged 18-24 years, we performed a multivariate analysis considering gender and age (Table 3). On the one hand, after multivariate analysis by gender in the group of women, students, eating disorder, and anxiety disorder remained with a significance at <.05 as diagnostic predictors of BDD (Table 3). In the male group after multivariate analysis, none of the factors analyzed showed statistical significance. On the other hand, in the multivariate analysis by age (Table 3), the following factors remained with significance at <.05 as diagnostic predictors of BDD. In the 18-24 years analysis, the diagnostic predictors were female gender and other gender, students, depressive disorder, eating disorder, and anxiety disorder. In the 25-44 years analysis, the diagnostic predictor was income level between €2000 and €2999. In the 45-64 years analysis, the diagnostic predictor was female gender, and the >64 years analysis was not performed due to the small sample size.

**Table 3:** Multivariate analysis by group.

			BDD^a^ prevalence, n/N (%)	Risk ratio (95% CI)	*P* value	Multivariate risk ratio (95% CI)	*P* value
**Female gender**							
	**Occupation**						
		Student	233/752 (30.9)	Ref^b^	—^c^	—	—
		Worker	43/541 (7.9)	0.19 (0.14-0.27)	.001	0.46 (0.22-0.96)	.04
		Other	9/128 (7)	0.17 (0.08-0.34)	.001	0.26 (0.09-0.72)	.01
	**Monthly income (€)^d^**						
		<500	181/617 (29.3)	Ref	—	—	—
		500-999	30/107 (28)	0.94 (0.59-1.48)	.79	—	—
		1000-1999	34/255 (13.3)	0.37 (0.25-0.55)	.001	—	—
		2000-2999	8/192 (4.2)	0.10 (0.05-0.22)	.001	0.37 (0.15-0.93)	.04
		>3000	91/173 (5.2)	0.13 (0.07-0.26)	.001	—	—
	**Eating behavior disorder**						
		Yes	69/125 (55.2)	6.195 (4.23-9.07)	.001	4.03 (2.32-6.98)	.001
		No	215/1296 (16.6)	Ref	—	—	—
	**Anxiety disorder**						
		Yes	103/250 (41.2)	3.83 (2.85-5.16)	.001	2.94 (1.65-5.25)	.001
		No	181/1171 (15.5)	Ref	—	—	—
**18-24 years age group**							
	**Gender**						
		Female	236/787 (30)	4.20 (2.75-6.42)	.001	3.19 (2.05-4.97)	.001
		Male	27/292 (9.2)	Ref	—	—	—
		Other	4/12 (33.3)	4.91 (1.39-17.37)	.01	6.38 (1.72-23.67)	.006
	**Occupation**						
		Student	255/1005 (25.4)	Ref	—	—	—
		Worker	8/67 (11.9)	0.40 (0.19-0.85)	.02	0.39 (0.17-0.86)	.02
	**Dermatologic disease**						
		Yes	177/640 (27.7)	1.53 (1.15-2.05)	.004	—	—
		No	90/451 (20)	Ref	—	—	—
	**Psychiatric disorder**						
		Yes	130/301 (43.2)	3.62 (2.70-4.86)	.001	—	—
		No	137/790 (17.3)	Ref	—	—	—
	**Depressive Disorder**						
		Yes	73/152 (48)	3.55 (2.49-5.06)	.001	1.71 (1.04-2.84)	.04
		No	194/930 (20.7)	Ref	—	—	—
	**Eating disorder**						
		Yes	66/107 (61.7)	6.27 (4.12-9.54)	.001	3.70 (2.13-6.42)	.001
		No	201/984 (20.4)	Ref	—	—	—
	**Anxiety disorder**						
		Yes	93/194 (47.9)	3.83 (2.76-5.30)	.001	2.14 (1.22-3.78)	.008
		No	174/897 (19.4)	Ref	—	—	—
**25-44 years age group**							
	**Monthly income (€)**						
		<500	9/35 (25.7)	Ref	—	—	—
		500-999	3/17 (17.6)	Ref	—	—	—
		1000-1999	15/109 (13.8)	Ref	—	—	—
		2000-2999	2/74 (2.7)	0.08 (0.02-0.40)	.002	0.12 (0.02-0.86)	.04
		>3000	1/43 (2.3)	0.07 (0.01-0.57)	.01	—	—
**45-65 years age group**							
	**Gender**						
		Female	21/403 (5.2)	6.76 (1.57-29.10)	.01	5.17 (1.14-23.45)	.03
		Male	2/248 (0.8)	Ref	—	—	—
	**Dermatologic disease**						
		Yes	16/273 (5.9)	3.33 (1.35-8.20)	.006	—	—
		No	7/381 (1.8)	Ref	—	—	—
	**Other dermatitis conditions**						
		Yes	3/13 (23.1)	9.31 (2.38-36.47)	.001	6.66 (1.44-30.87)	.02
		No	20/641 (3.1)	Ref	—	—	—

^a^BDD: body dysmorphic disorder.

^b^Ref: reference value.

^c^Not applicable.

^d^€1=US $1.11.

**Figure 3:**
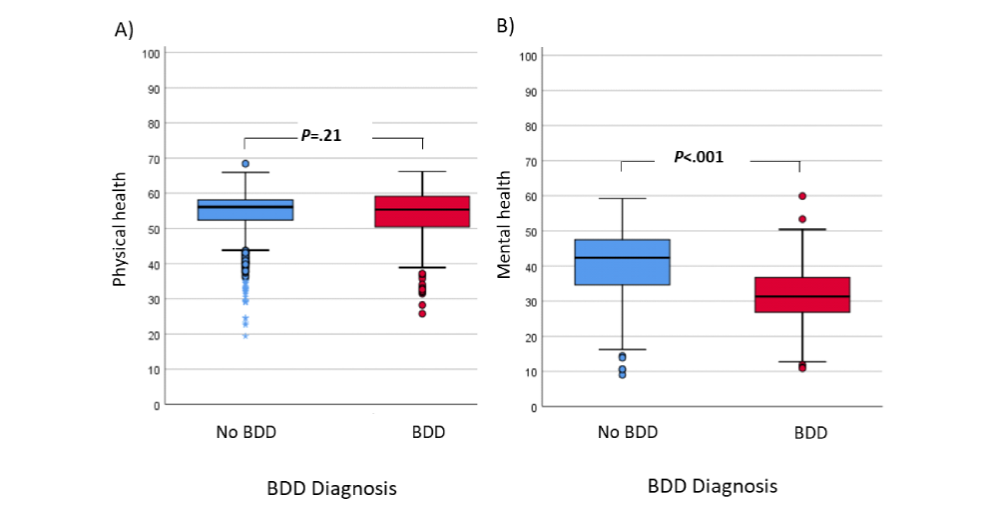
Association of body dysmorphic disorder with (A) physical and (B) mental health status. BDD: body dysmorphic disorder.

**Figure 4:**
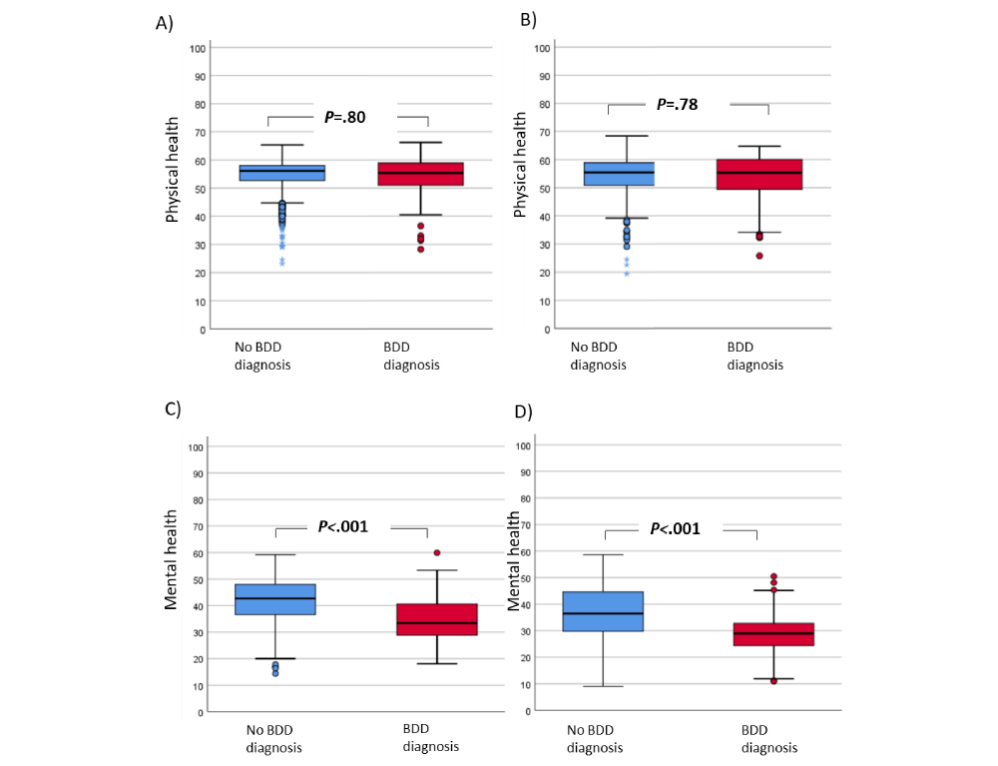
Perceived quality of life. (A) Physical health quality of life among patients without psychiatric comorbidities. (B) Physical health quality of life among patients with psychiatric comorbidities. (C) Mental health quality of life among patients without psychiatric comorbidities. (D) Mental health quality of life among patients with psychiatric comorbidities. BDD: body dysmorphic disorder.

Regarding quality of life, BDD was not statistically associated with physical health status. However, those diagnosed with BDD showed significantly lower levels of mental health than those without BDD (Figure 3). Additionally, quality of life analysis was performed considering psychiatric comorbidity as a factor affecting quality of life. Differences regarding mental health status remained statistically significant (*P*<.05) for patients with BDD, irrespective of the presence of psychiatric pathology (Figure 4).

## Discussion

### Principal Findings

Our study reveals many interesting aspects of BDD. Most of our findings are consistent with those reported previously [1,4,6,12,13]. For example, BDD is particularly prevalent in the young adult population [5], and patients with BDD are particularly concerned about an average of 4.6 different body parts. However, some findings are novel in our study. For instance, the prevalence of BDD in our sample population (15.2%) was higher than expected (0.5%-3.2%) [1]. Further, in addition to the described association between BDD and eating and depressive disorders, we report that BDD is closely associated with anxiety disorders. The most relevant finding of our study is the perception of quality of life: participants with BDD had a poorer perception of quality of life associated with mental but not physical health problems. Moreover, the perception of quality of mental health in patients with BDD was independent of diagnosis of any mental disorder.

Our study reports that the prevalence of BDD in adults is 15.2% in Spain, which is higher than that reported in the general population (0.5%-3.2%) in another study [1] and higher than that reported in a Spanish multicentric study in patients with acne (10.6%) [2]. Our findings may be explained by 2 factors. First, our sample population was particularly young, with more than half of the participants being in the age group of 18-24 years. As shown in a previous study [6], the younger the age of the patient, the greater is the possibility of BDD diagnosis. Second, our data reflect more of a screening diagnosis as compared with a definitive diagnosis established with BDDQ via an interview by a health professional, which is more demanding [17].

Our study shows that the sociodemographic characteristics most associated with the diagnosis of BDD are gender (female and other), age group of 18-24 years, students, income level of less than €500/month, and a diagnosis of previous dermatologic or psychiatric disease. Regarding gender, females showed a statistically significant (*P*<.001) higher prevalence of BDD than males (284/1421, 19.9% vs 34/653, 5.2%, respectively). Previous studies have reported similar prevalence between men and women [3] or increased prevalence in women (68.5%) compared to that in men [4]. In our study, almost 67.9% (1421/2091) of the participants were women, which may suggest the need for future studies controlled by sex to clarify the differences. Regarding age, the mean age at diagnosis of BDD in the participants in our study was 23.5 years, which is higher than the mean age of 16.9 years described elsewhere [5]. This is probably because our study only included populations 18 years and older, resulting in an increase in the mean age at diagnosis. Furthermore, we found a higher prevalence of BDD among students (263/1043, 25.2%), which was also higher than 1.3%-5.8% reported in another study [1]. Again, this could be because almost half our sample was comprised of students. Moreover, this can be attributed to the influence of social media in the current age, increasing young people's concern about their body image [1]. It would be appropriate to conduct a more specific study including this young population group.

Participants with BDD in our study were concerned about an average of 4.6 different body parts, which is in line with that reported by previous studies (5-7 body parts) [13]. The body parts that were of the most concern were body fat (248/322, 77%), thighs (191/322, 59.3%), face (166/322, 51.6%), hip (144/322, 44.7%), and skin (126/322, 39.1%). Skin (14/26, 54%) and face (8/26, 31%) were the body parts of the greatest concern in previous studies [1]. The prevalence of BDD in participants with dermatologic conditions (204/1072, 19%) in our study falls within the range (4.9%-21.1%) reported in the literature [1]. However, it is necessary to emphasize that previous studies did not distinguish between dermatologic patients per se and those undergoing aesthetic procedures [1].

Regarding the association between previous dermatologic disease and BDD, we found no significant relationships. The association between BDD and acne (115/532, 21.6%) in our study was slightly higher than that previously described (8.8%-21.1%) [1]. The association of BDD with having undergone rhinoplasty was 12.1% (39/322), which was lower than that previously documented (20.1%) [1]. However, the sample size limited our capability to extract meaningful conclusions on this issue. Among participants with previous psychiatric pathology and BDD, there was a significant association with eating disorders (72/136, 52.9%) and depressive disorders (84/244, 34.4%), similar to the findings of 12%-45% and 47%-56.3%, respectively, reported in a previous systematic review [1]. In addition, our study showed a significant comorbidity with anxiety disorders (108/292, 36.9%), making it necessary to conduct future studies in this subgroup.

Regarding quality of life, participants diagnosed with BDD had a perception of having a poorer mental health status than those without BDD. In contrast, there were no significant differences in the physical status between participants with and without BDD [12]. Ultimately, the diagnosis of BDD was associated with a perception of reduced quality of life that is not subsidiary to the presence of mental health disorders. In other words, our study suggests that BDD could be used as a marker or predictor of an individual's perception of quality of life, which is independent of the presence of mental health problems.

### Limitations and Strengths

Our study was based on the use of a questionnaire that was disseminated telematically, which is why we obtained a heterogeneous participant base, being represented mostly by female students in an age range of 18-24 years. The higher percentage of prevalence obtained may be linked to the specific population in this study. However, we must bear in mind that, despite obtaining the first diagnostic approximation with BDDQ, its confirmation must subsequently be backed up by an interview with a health professional [17]. Furthermore, the mean age at BDD diagnosis (23.5 years) in our study was higher than that (16.9 years) described previously [5] because this study was limited to participants aged 18 years and older. Additionally, the number of participants in our study who had undergone previous plastic surgeries was too less to achieve a proper statistical power.

With regard to the strengths of this study, we increased the number of variables related to BDD compared to the number of variables used in previous studies [2-6,12,13], which, together with the total number of participants, resulted in a large database. Further, we introduced the quality of life indicators through the SF-12v2 health survey.

### Conclusion

Patients with BDD experience serious biopsychosocial repercussions. This study provides the first approximation of the prevalence of BDD in the Spanish population, which was found to be higher than expected, although our results should be interpreted cautiously. BDD was particularly prevalent in participants aged 18-24 years, students, and women. BDD is associated with psychiatric conditions such as eating disorder, anxiety disorder, and depressive disorder, and with dermatologic conditions such as acne and dermatitis. No significant associations were found between BDD and the performance of previous aesthetic procedures, which could be due to our small sample size. Finally, BDD could be a marker of an individual’s perception of quality of health, irrespective of psychiatric diagnoses. Future studies should confirm our preliminary findings.

## Abbreviations

BDD: body dysmorphic disorder
BDDQ: Body Dysmorphic Disorder Questionnaire
SF-12v2: 12-item Short Form version 2
